# Corrigendum to: Totally endoscopic aortic valve replacement via antero-lateral approach using a standard prosthesis

**DOI:** 10.1093/icvts/ivac048

**Published:** 2022-02-28

**Authors:** Masayoshi Tokoro, Sadanari Sawaki, Takahiro Ozeki, Mamoru Orii, Akihiko Usui, Toshiaki Ito

**Affiliations:** 1 Department of Cardiovascular Surgery, Japanese Red Cross Nagoya First Hospital, Nagoya, Japan; 2 Department of Cardiac Surgery, Nagoya University Graduate School of Medicine, Nagoya, Japan

[Interact CardioVasc Thorac Surg 2020; https://doi.org/10.1093/icvts/ivz287]

In the originally published version of this manuscript, there was an error in the affiliation of corresponding author Masayoshi Tokoro. At the time of publication, the author was affiliated to both “Department of Cardiovascular Surgery, Japanese Red Cross Nagoya First Hospital, Nagoya, Japan” and “Department of Cardiac Surgery, Nagoya University Graduate School of Medicine, Nagoya, Japan”.

